# Real-world data for the availability of pediatric medicines in Chinese hospitals: a multi-center survey and analysis

**DOI:** 10.3389/fphar.2024.1283068

**Published:** 2024-02-14

**Authors:** Wen-Yi Ruan, Hui-Ying Chen, He-Ping Cai, Xiao-Ling Wang, Zhi-Gang Zhao

**Affiliations:** ^1^ Anhui Provincial Children’s Hospital, Department of Clinical Pharmacy, Hefei, China; ^2^ National Center for Children’s Health, Department of Pharmacy, Beijing Children’s Hospital, Capital Medical University, Beijing, China; ^3^ Department of Pharmacy, Beijing Tiantan Hospital, Capital Medical University, Beijing, China

**Keywords:** pediatric, medicine classification, availability, multi-center survey, real-world data

## Abstract

**Aim:** No information exists on the availability of pediatric medicines in China. This study aimed to access the availability of different types of pediatric medicine and determine their ratio in medical institution drug catalogs.

**Methods:** Based on drug instructions, an expert meeting method was used to divide pediatric medicines into five categories: child-specific medicine (CSM), co-use medicine for adults and children (CMAC), other pediatric medicines (OCM), off-label medicine use (OMU), and non-child medicine (NM).

**Results:** A total of 60 hospitals nationwide participated in this survey, namely, 20 children’s hospitals (C-hosp), 14 maternal and child healthcare hospitals (MCHC-hosp), and 26 general hospitals (G-hosp). The average number of drug catalogs in G-hosp was significantly higher than that in C-hosp and MCHC-hosp. CSM accounted for 9.77% of the C-hosp catalog, 7.12% of the MCHC-hosp catalog, and 1% of the G-hosp catalog. The availability rate of CMAC was 49.63% in C-hosp and 40.87% and 31% in MCHC-hosp and G-hosp, respectively. The proportion of OCM in C-hosp (27.28%) was higher than that in MCHC-hosp (13.4%) and G-hosp (5%). The OMU occupied ratio in C-hosp, MCHC-hosp, and G-hosp is not negligible, which was 12.06%, 8.7%, and 10% respectively. The proportion of NM in C-hosp was almost negligible but was 29.91% and 53% in MCHC-hosp and G-hosp, respectively. Compared to the CSM and CMAC listed in China, the share of CSM in C-hosp was close to 40%, which was much higher than that of G-hosp and MCHC-hosp. In contrast, the share of CMAC in G-hosp was nearly 30%, which was significantly higher than that in C-hosp and MCHC-hosp. Health insurance covers most of these five types of pediatric medicines, with the proportion of insured medicines reaching close to 80% in C-hosp and approximately 85% in MCHC-hosp and G-hosp.

**Discussion:** The availability of specific medicines suitable for use in children is generally low, and even CSM in specialized hospitals such as C-hosp cannot meet the relatively high accessibility level of WHO evaluation standards. Policies and measures should be implemented to boost the research and development of pediatric medicines, as well as supplement safety information lacking in instruction manuals.

## 1 Introduction

The differences in physiology between adults and children make it difficult to define them as “small adults,” meaning that differences in pharmacokinetic characteristics between the two have been observed for many drugs (CHU et, al. 2022). The specificity of pediatric medicines in terms of safety, type, dosage, taste, etc., makes adult drugs not directly applicable to children (Rui-lin, et, al. 2023). Off-label drug use is common in pediatric clinics. Previous studies have shown that although American pediatricians prescribe more than 40 million off-label prescriptions annually, an increasing trend is still observed year by year (Hoon et, al. 2019; Tang et, al. 2023). The results showed that the proportion of off-label drug use in pediatric patients was 46.5%, whereas that of unauthorized drug use was 11.4% (Wang Y. 2018). Previous statistical analyses have shown that the current situation of children’s medication use has few specifications of special dosage forms, lack of varieties, unclear information about children’s instructions, and off-label drug use (Kesti et, al. 2023; Chen, et, al. 2021). Accordingly, the availability of medicines for pediatric patients is currently the focus of national and pediatric clinical attention. In recent years, the government has frequently released key policies on pediatric medicine.

To comprehensively assess the distribution and proportion of pediatric medications in Chinese medical institutions, we categorized them into five distinct groups: child-specific medication (CSM), medications suitable for both adults and children (CMAC), other pediatric medicines (OCM), off-label medication use (OMU), and non-pediatric medications (NM). Following this, we conducted a nationwide survey on the accessibility of real-world data. The analysis and discussion of the real-world data obtained provide the basis for better development and policy research on pediatric medicines.

## 2 Methods

### 2.1 Classification and definition of pediatric medicines

After the expert meeting discussion, pediatric medicines were categorized into five groups primarily based on the information provided on their drug labels. These five categories of drugs are defined as follows:

1. CSM: The medicine specification only contains information about children’s medication without adult medication and clear usage, dosage, and safety information related to children’s medication.
2. CMAC: The medicine specification covers both children and adult medication information and has clear usage and dosage related to children, as well as safety knowledge.
3. OCM: The medicine specification has relevant information on children’s medication, but the use and dosage of children’s medication are summarized as “discretionary” or “according to the doctor’s advice,” which cannot be clearly used.
4. OMU: In special cases where there is no effective or better treatment, physicians follow the clinical application guiding principles, clinical diagnosis, and treatment guidelines, etc., to use medicines that are not specified in the instructions but are evidence-based medicine.
5. NM: Refers to medicines that are not within the definition of the above types and are not clinically used for children (such medicines are mainly for some in the drug catalog of general hospitals or maternal and child health hospitals).

### 2.2 Descriptive analysis study and survey participants

This study is mainly descriptive of the drug catalog provided by hospitals nationwide. The catalogs we received included information about the drug’s generic name, the specification of the dosage form, the manufacturer, whether it is covered by medical insurance, and the type of children’s medication. Based on these drug catalogs from hospitals, information on the availability of different types of pediatric medicines can be obtained. Availability refers to the percentage of hospitals surveyed that have access to a drug. The purpose of this survey was to determine the proportion and accessibility status of different types of pediatric medicine in the drug catalogs of children’s hospitals (C-hosp), maternal and child healthcare hospitals (MCHC-hosp), and general hospitals (G-hosp). According to the World Health Organization (WHO) and Health Action International (HAI) evaluation standards, availability <30% is very low, 30%–49% is considered low, 50%–80% is relatively high, and >80% is high (WHO, 2006).

From August 12 to 24 August 2022, the status investigation method was used to collect the in-hospital drug catalog and types of pediatric medicine from 60 medical institutions nationwide using a questionnaire. The hospitals involved in the survey were primarily C-hosp, MCHC-hosp, and G-hosp.

### 2.3 Regional distribution of investigated hospitals

Research on C-hosp and MCHC-hosp was based on the Futang Research Center of Pediatric Development. The area covers seven regions: Central China, East China, North China, Northwest, Northeast, South China, and Southwest. It covers 20 provinces (Hebei, Shanxi, Jiangsu, Zhejiang, Anhui, Shandong, Fujian, Jiangxi, Henan, Hubei, Hunan, Shanxi, Gansu, Qinghai, Heilongjiang, Jilin, Guangdong, Hainan, Yunnan, and Guizhou), 4 autonomous regions (Ningxia, Xinjiang, Neimenggu, and Guangxi), and 4 municipalities (Beijing, Shanghai, Tianjin, and Chongqing). According to the 7th national census data on the National Bureau of Statistics, the population of children aged 0∼14 years in the research area accounted for 92.46% of the overall child population nationwide.

### 2.4 Data analyses

The data were analyzed descriptively and tabulated.

## 3 Results

### 3.1 Investigated provinces and distribution of hospitals

**FIGURE 1:**
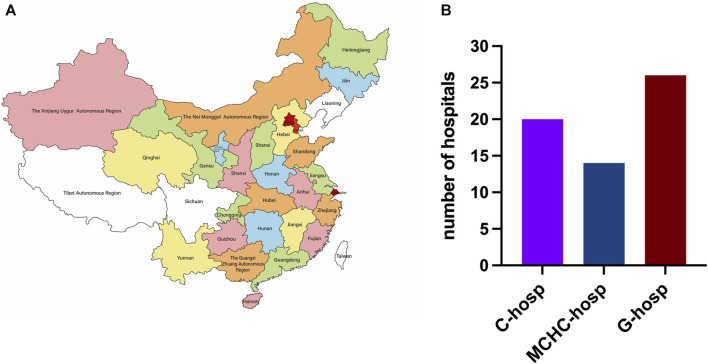
Distribution map of provinces **(A)** and medical institutions surveyed **(B)**.

The distribution of provinces in this multicenter survey is shown in Figure 1A, including 20 provinces, 4 municipalities, and 4 autonomous regions. Only 3 provinces were not covered because they were in the period of COVID-19 prevention and control at that time and failed to respond in time to the questionnaire. A total of 60 hospitals were surveyed, namely, 20 C-hosp, 14 MCHC-hosp, and 26 G-hosp, as shown in Figure 1B.

### 3.2 The average number of drug varieties in medical institutions

**FIGURE 2:**
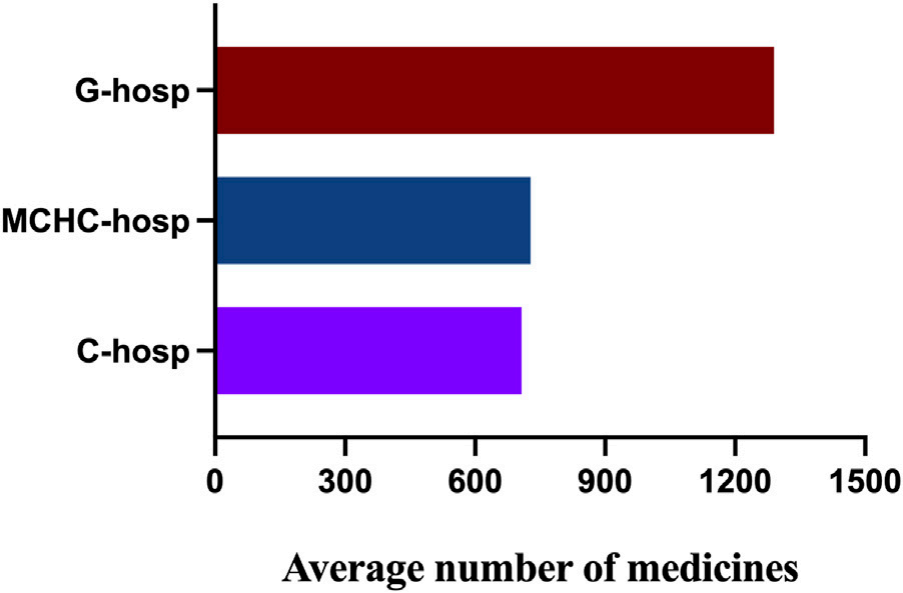
Average number of drugs in C-hosp, MCHC-hosp, and G-hosp.

The results of the average number of varieties in C-hosp, MCHC-hosp, and G-hosp gained after the investigation are shown in Figure 2. The average number of drugs in the C-hosp and MCHC-hosp was similar, while that in the G-hosp was much higher.

### 3.3 Proportion of pediatric medicine types in medical institutions

**FIGURE 3:**
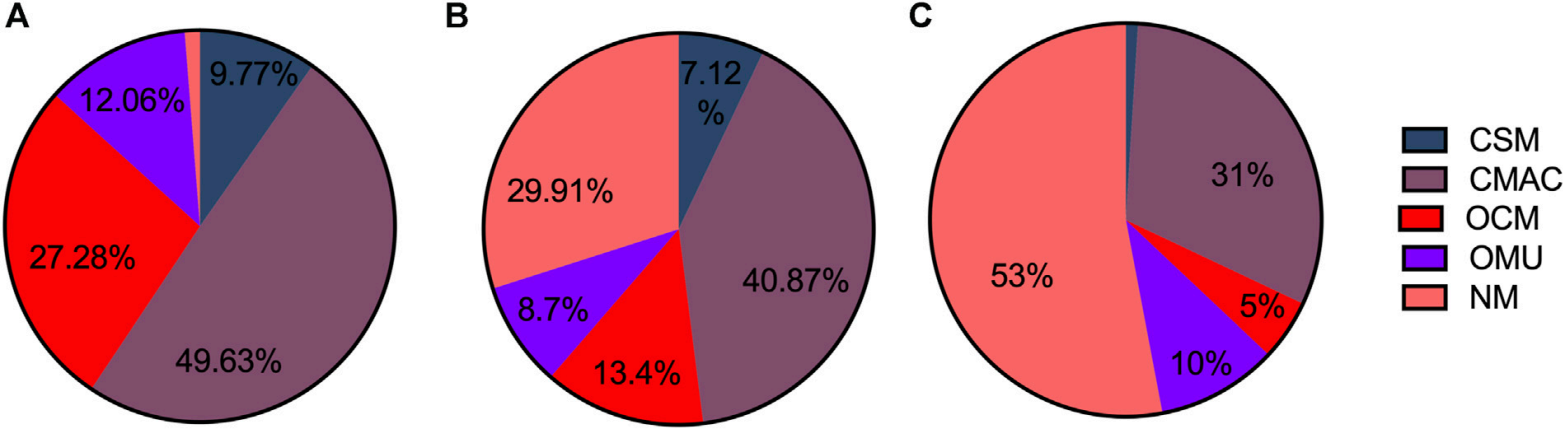
Proportion of different pediatric medicine types in C-hosp **(A)**, MCHC-hosp **(B)**, and G-hosp **(C)**.

The proportion of different types of pediatric medicines in C-hosp is shown in Figure 3A. The CSM proportion was 9.77%, whereas the CMAC proportion was the highest but no more than 50%, followed by OCM, OMU, and NM. Due to the specificity of C-hosp, the proportion of NM was only 1.26%, which is in line with reality. The occurrence of non-pediatric drugs in C-hosp may be due to some adult departments in some C-hosp, such as gynecology and obstetrics.

The proportion of different types of pediatric medicines in MCHC-hosp is shown in Figure 3B. CSM accounted for 7.12%, with CMAC having the highest proportion, followed by OCM, OMU, and NM.

The proportions of different types of pediatric medicines in G-hosp are shown in Figure 3C. The largest proportion was NM, which exceeded 50%, followed by CMAC, OCM, and OMU. CSM accounted for the smallest proportion (only 1%). The investigation results are basically in line with the actual situation that the proportion of pediatric medicines in the drug catalog of G-hosp is relatively small.

### 3.4 Comparison of CSM in medical institutions

**FIGURE 4:**
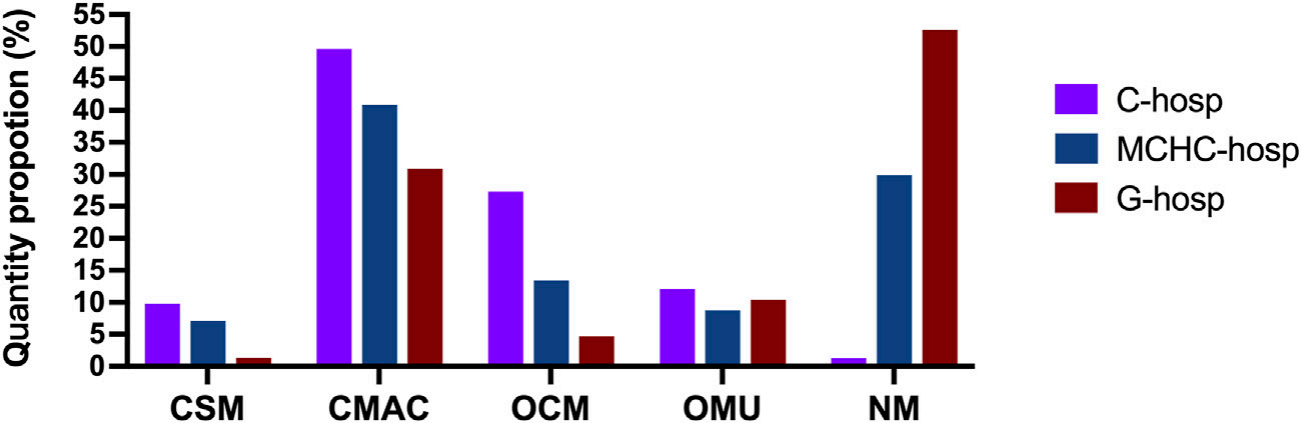
Proportion of different pediatric medicine types in medical institutions.

**FIGURE 5:**
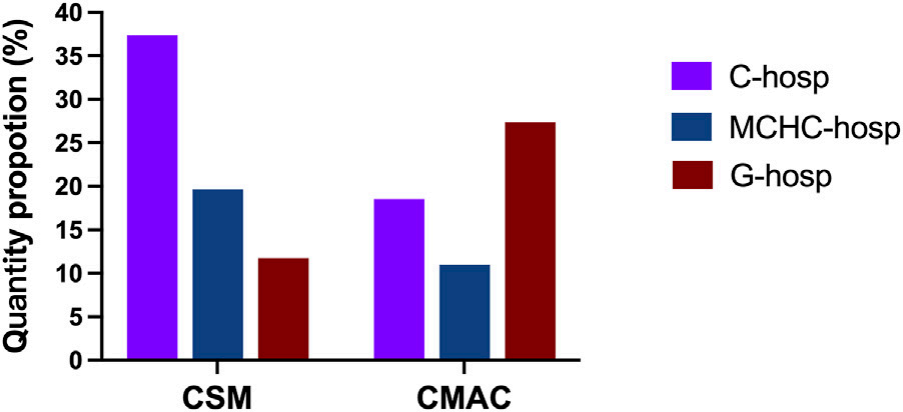
Available rate of CSM and CMAC in medical institutions.

As shown in Figure 4, the proportion of CSM in C-hosp was higher than that in MCHC-hosp and G-hosp. However, even in C-hosp, the ratio of CSM was less than 10%, which means that availability was very low. Based on the Chinese Pediatric Drug Database, the number of CSM in the market in China by August 2022 was 3,695. Therefore, the availability of CSM in medical institutions can be calculated (Figure 5). The available rate of CSM in the C-hosp was close to 40%, which is much higher than that in MCHC-hosp and G-hosp. The available rate in G-hosp was only approximately 10%.

### 3.5 Comparison of CMAC in medical institutions

The proportion of CMAC in the drug catalog of C-hosp, MCHC-hosp, and G-hosp is shown in Figure 4. Similar to CSM, the proportion of CMAC in C-hosp was higher than that in MCHC-hosp and G-hosp. It can be clearly seen that the proportion of CMAC in C-hosp reached nearly 50%. Compared with other types of pediatric medicines, CMAC occupied the most significant proportion of C-hosp and MCHC-hosp. It is worth mentioning that the CMAC proportion in G-hosp was only lower than that of NM. According to the Chinese Pediatric Drug Database, the number of CMAC in the market in China by August 2022 was 37,877. So, the availability of CMAC in medical institutions could be calculated (Figure 5). It can be seen that the ratio of CMAC availability in G-hosp was higher than that in C-hosp and MCHC-hosp, but the quota was less than 30%, indicating very low availability.

### 3.6 Proportions of OCM, OMU, and NM in medical institutions

As shown in Figure 4, the proportion of OCM in C-hosp was obviously higher than that in MCHC-hosp and G-hosp, reaching 27.28%. The difference between the proportions of OMU in the C-hosp, MCHC-hosp, and G-hosp was not obvious. For NM, due to the particularity of G-hosp and MCHC-hosp, which have many adult departments, the proportion of NM in them was much higher than that in C-hosp. In particular, in G-hosp, the proportion of NM was as high as 52.61%.

### 3.7 Proportion of medical insurance drugs in pediatric medicine

The proportion of medical insurance medicines in the hospital drug catalog of MCHC-hosp and G-hosp reached approximately 85%, and that of C-hosp reached approximately 80%, which was slightly lower (Table1). It can be seen that medical insurance medicines are highly available in medical institutions.

**TABLE 1:** Proportions of medical insurance medicines and non-medical insurance medicines in C-hosp, MCHC-hosp, and G-hosp.

Medical institution	Medical insurance medicine (%)	Non-medical insurance medicine (%)
C-hosp	79.67	20.33
CMHC-hosp	85.6	14.4
G-hosp	85.81	14.19

The proportions of different types of pediatric medicines covered by medical insurance in medical institutions are shown in Table 2. In addition to the relatively low medical insurance coverage proportion of NM in the MCHC-hosp, the rest basically accounted for more than 60%. Thus, we can conclude that the current availability of Medicare medicines for these five pediatric medicine types is high.

**TABLE 2:** Proportions of different pediatric medicine types covered by medical insurance in medical institutions.

Type	C-hosp (%)	CMHC-hosp (%)	G-hosp (%)
CSM	75.64	68.58	78.40
CMAC	88.43	97.46	84.57
OCM	85.31	99.14	86.71
OMU	84.73	77.77	92.06
NM	76.40	18.45	86.00

## 4 Discussion

Guaranteeing children’s medications is a universal problem worldwide. The investigation and analysis of the drug catalogs of C-hosp, MCHC-hosp, and G-hosp in China showed that G-hosp exhibits a considerably higher quantity of drugs than specialized hospitals such as C-hosp and MCHC-hosp. This finding aligns with the prevailing circumstances. In addition, the results of this survey also reflect several phenomena that are outlined as follows. First, combined with the current drug supply and clinical use, there still needs to be more suitable medicines for pediatrics. However, there is a significant scarcity of CSM. Even in C-hosp and G-hosp, the proportion of CSM was only 9.77% and 7.12%, respectively, which was far lower than the WHO standard of low availability of medicines. It shows that there are few suitable specifications, dosage forms, and varieties of pediatric medicines for pediatric clinics to select. Second, CSM was in short supply nationwide. According to the data from the Chinese Pediatric Drug Database, the CSM in C-hosp only accounted for 40% of the market in China, while the data for MCHC-hosp and G-hosp were only approximately 20% and 10%, respectively. These data illustrate that the lack of CSM for pediatric clinics may be partly due to the low CSM proportion in the drug catalog of medical institutions, which then leads to less selectivity in pediatric clinical drug choices. Third, there is a lack of information regarding pediatric medication. Many medicines, such as OCM and OMU, have little information about pediatric medication in the drug instruction. Therefore, clinicians rely more on empirical or off-label drug use during the therapeutic process. The proportion of OCM and OMU in the C-hosp was close to 40%, which should not be ignored. Although its proportion in MCHC-hosp and G-hosp was 22.1% and 15%, respectively, it was significantly lower than that of C-hosp. This may be due to the fact that pediatric medicines in MCHC-hosp and G-hosp are not the main components of their hospital drug catalogs, which does not mean that empirical drugs and off-label drugs are rare in their pediatric clinical use. Fourth, there is healthcare coverage. The accessibility of Medicare medicine in C-hosp, MCHC-hosp, and G-hosp reached 80% or more, indicating a high availability rate.

## 5 Limitations

The study was a multi-center investigation that only collected drug catalogs currently listed by provincial medical institutions. The first limitation was that prefecture-level city hospitals and community clinics were not surveyed. Therefore, the results of this study only describe the current state of pediatric medicine in general rather than in detail. The second limitation was that this study sorted out and analyzed the drug catalogs of some hospitals and lacks sorting and analysis of the types of drugs currently listed in China.

## 6 Conclusion and suggestion

This study shows the current availability and proportion of pediatric medicines in Chinese medical institutions. Based on the results of this study, to better improve the availability of pediatric medicines in medical institutions, especially in C-hosp, the following suggestions were made: 1) a pediatric medicine review group and a pediatric drug plan applicable to the current situation should be established to optimize and shorten the approval process for CSM. Japan’s Ministry of Health, Labour, and Welfare (MHLW) set up a pediatric medicine group in 2011 to strengthen cooperation and information exchange on pediatric medicine at home and abroad (Hong et, al. 2022). The US Food and Drug Administration (FDA) and the European Medicines Agency (EMA) proposed pediatric research plans for “IPSP” and “PIP” in 2012 and 2006, respectively (Ning, et, al. 2022; Wang et, al. 2021). These plans aim to conduct the approval of pediatric medicines in a timely manner, optimize and simplify the approval process, and provide it a patent extension or market protection period and other corresponding incentive policies. Implementing these policies is conducive to encouraging the enthusiasm for research and development of pediatric medicine and speeding up the listing process; 2) it is recommended that research, development, and innovation mechanisms are encouraged. From a policy perspective, incentives should be increased for the research and development of pediatric medicine dosage forms and specifications to resolve problems such as unstandardized drug usage (Wang et, al. 2023). According to previous statistics, a total of 39 documents related to pediatric medicine use were collected at the national level from January 2010 to December 2021 (Rong et, al. 2022; Zhang et, al. 2023). Although relevant documents are issued every year, the frequency of publications has gradually increased in recent years. However, it still cannot well meet the policy needs for developing pediatric medicine use; and 3) medical institutions should be encouraged to research pediatric medicine use and collect and analyze data related to pediatric medicine. This is because it fills the gap in the data informatization of pediatric medicine applications.

## Data Availability

The original contributions presented in the study are included in the article/Supplementary Material; further inquiries can be directed to the corresponding author.
